# Preparation and in vivo bacteriostatic application of PPDO-coated Ag loading TiO_2_ nanoparticles

**DOI:** 10.1038/s41598-022-14814-6

**Published:** 2022-06-22

**Authors:** Tongyan Ren, Chengmin Feng, Jun Dong, Hong Zhu, Bing Wang

**Affiliations:** 1 Department of Chemistry, School of Pharmacy, North Sichuan Medical College, Nanchong, China; 2 Department of Otorhinolaryngology and Head & Neck Surgery, Affiliated Hospital of North Sichuan Medical College, Nanchong, China; 3 Department of Immunology, School of Basic Medicine and Forensic Medicine, North Sichuan Medical College, Nanchong, China; 4 Medical Imaging Key Laboratory of Sichuan Province, North Sichuan Medical College, Nanchong, China

**Keywords:** Biomaterials, Polymers

## Abstract

Implant-associated infections limit the clinical application of implants therapy; hence, exploiting strategies to prevent biomaterial-associated infections has become important. Therefore, in this study, a series of poly (p-dioxanone) (PPDO)-coated Ag loading TiO_2_ nanoparticles (Ag@TiO_2_-PPDO) was synthesized to be applied as bacteriostatic coating materials that could be easily dispersed in organic solvent and coated onto implantable devices via temperate methods such as electrospraying. The lattice parameters of TiO_2_ were a = 0.504 nm, b = c = 1.05 nm, alpha = beta = gamma = 90 degree and the size of crystallite was about 13 nm, indicating that part of Ag has been embedded into crystal defects of TiO_2_. Both XRD and TEM determinations indicated the successful grating of PPDO on the surface of Ag@TiO_2_. Among Ag@TiO_2_ nanoparticles with various Ag loading quantities, 12% Ag@TiO_2_ nanoparticles exhibited relatively higher grafting efficiency and Ag contents on the surface of grafted composites. In addition, 12% Ag@TiO_2_-PPDO exhibited the best bacteriostatic effect in vitro owing to its higher grafted efficiency and relatively short length of PPDO segments. Subsequently, Ag@TiO_2_-PPDO was coated on the surface of a poly lactic-co-glycolic acid (PLGA) electrospun membrane via the electrospraying method. Finally, the in vivo bacteriostatic effect of 12% Ag@TiO_2_-PPDO coating was verified by implanting 12% Ag@TiO_2_-PPDO-coated PLGA membrane into a rat subcutaneously combined with an injection of *Staphylococcus aureus* at implanting sites.

## Introduction

With the development of medical and material science and technology, implantable biomaterials with variable functions have been widely adopted in various applications such as oral cavity, abdomen, and orthopedics^1–3^. However, implant-associated infections pose a limitation to implant therapy in clinical practice^4–6^. Such infections may lead to therapy failure, secondary surgery, and even disabilities^7–10^. Except for economic burden, these complications would also cause additional pain and suffering to patients^11^.

Therefore, exploiting different strategies to prevent biomaterial-associated infections has garnered considerable attention^12–14^. These include systemic applications of antibiotics after surgery and the surface modifications of implantable devices to make them robust against bacterial adhesion or prevent bacterial colonization of the device upon implantation^15,16^. However, systemic applications of antibiotics might trigger antibiotic resistance. Hence, it is essential to modify implants and endow them with spontaneous bacteriostatic properties^17^. Among numerous modification strategies, Ag coating has garnered the interest of several researchers, owing to its low toxicity and long-term antimicrobial effect against gram-negative bacteria, fungi, protozoa, and certain viruses^18^. Ag-contained formulations include Ag salts, Ag oxide, chelated Ag, or Ag nanoparticles^19^.

TiO_2_ nanoparticles (TiO_2_ NPs) could function as an Ag carrier for the antimicrobial modification of biomaterials. TiO_2_ can decompose microorganisms in the presence of ultraviolet (UV) lights because of its photocatalytic effect; hence, it could be applied as biofunctional coating in preoperative sterilization and self-cleaning of implanted materials^20,21^. The addition of noble metals such as Ag to TiO_2_ and forming composite structures could enhance absorption in the visible light range^22^. Ag can be embedded into TiO_2_ lattice such as during hydrothermal processes because titanate nanostructures possess large surface area and more defects^23^. Otherwise, Ag could also deposit with wide size-distribution on the surface of the individual TiO_2_ crystallites^24^. Incorporation of Ag lowers the bandgap of photogenerated electron transition, effectively restrains the recombination of photogenerated electrons and holes, and further enhances the antibiotic efficiency of TiO_2_. Therefore, Ag@TiO_2_ NP coating devices such as Ag@TiO_2_ NP coated Ti mesh or polymeric membranes have been applied in biomedical fields such as hard tissue replacement to reduce post-implantation infection^25–28^.

Furthermore, there are several available methods for preparing Ag coating, including plasma sprayed^29^, electrochemically deposition^30^, sol–gel^31^, thermal spray^32^, co-sputtering^33^, and plasma immersion ion implantation methods^34^. However, these methods often require complex equipment and operation. In particular, biodegradable implantable membrane materials and implantable nonmetallic materials with low mechanical strength could be unable to bear intense environmental variations. Hence, it is necessary to investigate temperate coating strategies.

Electrospraying, a liquid atomization-based technique, involves the application of a high voltage to a polymer solution, which forces it to emerge from a syringe in the form of micro or nanoparticles, and is a convenient and efficient method for constructing nanoarchitectures and coating devices surfaces^35^. Electrospraying is a compatible method for coating device surfaces, as it could protect devices from high temperature, humidity, acid, and alkali environment, and is suitable for coating environment-sensitive implantable biomaterials.

**Figure 1:**
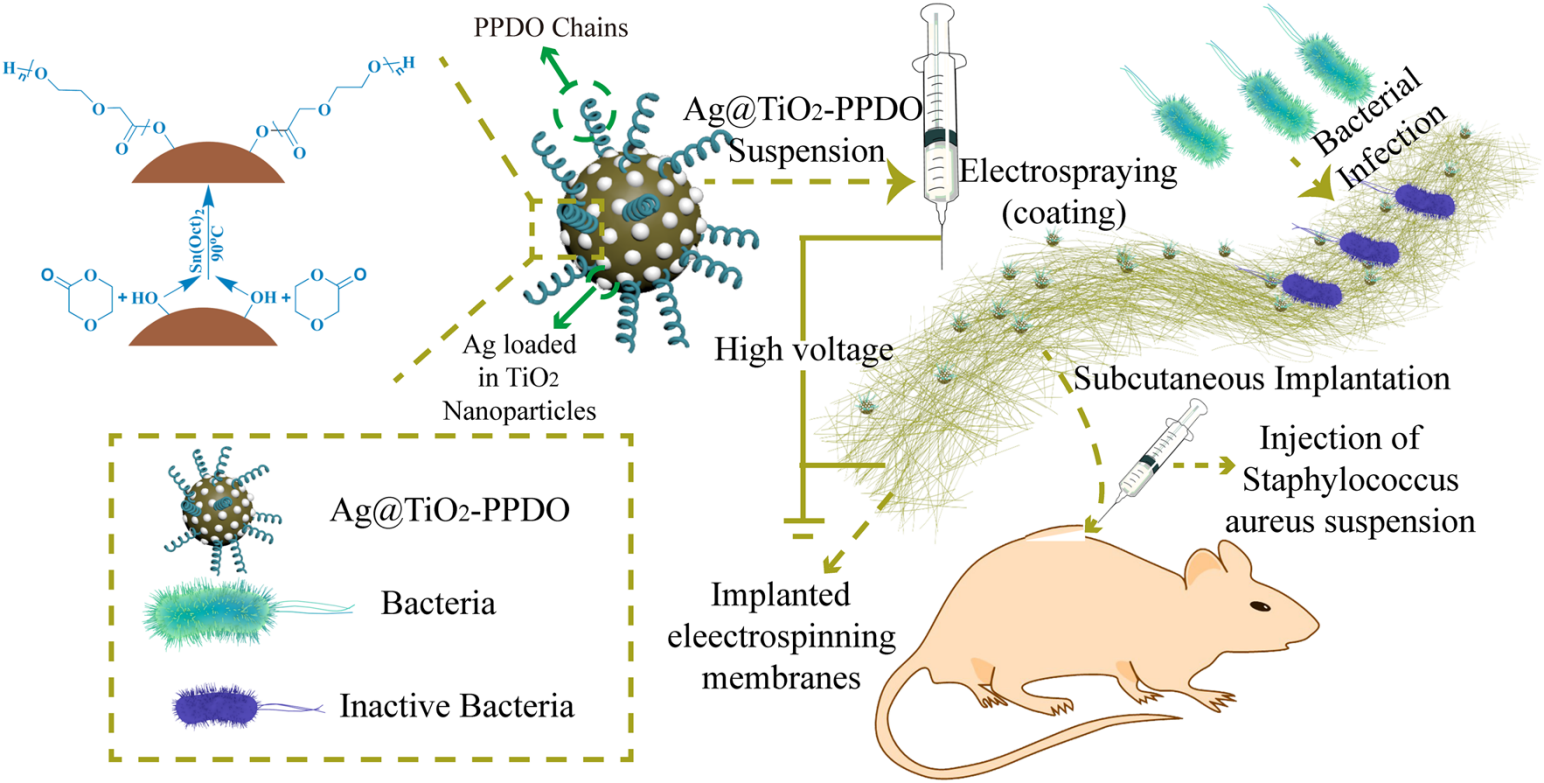
Synthetic processes of Ag@TiO_2_-PPDO and Ag@TiO_2_ NP-coated PLGA/PLCA electrospinning membrane.

In this research, we prepared an Ag@TiO_2_ NP-coated PLGA/PLCA electrospinning membrane via the electrospraying method to endow this membrane with antibacterial functions (Fig. 1). Because Ag@TiO_2_ nanoparticles could not be dissolved and dispersed uniformly in an organic solvent, poly p-dioxanone (PPDO) was first grafted onto Ag@TiO_2_ nanoparticles to form organic solvent-dispersive Ag@TiO_2_-PPDO nanoparticles. Subsequently, the antibacterial effect of Ag@TiO_2_-PPDO nanoparticles was investigated in vitro. Finally, Ag@TiO_2_-PPDO nanoparticles were coated onto PLGA/PLCA electrospinning membranes via electrospraying. The in vivo antibacterial effect of Ag@TiO_2_-PPDO nanoparticles was also verified by the infective subcutaneous implanting experiments of rats.

## Materials and methods

### Materials

All chemicals used in this research are commercially available and used without further purification. Specifically, nano-TiO_2_ (n-TiO_2_, anatase, 10-25 nm) and AgNO_3_ were purchased from Aladdin Biochemical Technology Co. Ltd. (China) and Chengdu Chron Chemicals Co. Ltd. (China), respectively. Nutrient agar, peptone, and yeast extract were purchased from Aobox biotechnology Co. Ltd. (China) and Oxoid (UK), respectively. *Staphylococcus aureus* was provided by the pathogen center of the North Sichuan Medical College. In total, 18 male SD rats (220 ± 20 g) were provided by the laboratory animal center of the North Sichuan Medical College (Nanchong, China). The rats were individually housed under pathogen-free conditions with free access to food and water. A hematoxylin and eosin (H&E) staining kit was purchased from Beyotime (Beijing, China), while the tumor necrosis factor alpha (TNF-α) monoclonal antibody for immunofluorescence were purchased from Abcam (UK).

### Characterizations

X-ray diffraction (XRD) spectra of Ag@TiO_2_-PPDOs were recorded on a D8 Advance X-ray diffractometer (Bruker, Germany) using Cu Kα radiation (λ = 1.5406 Å) to measure the phase structure. Then the XRD data of Ag@TiO_2_ NPs was analyzed by JADE Intelligent XRD Analysis Software (Ver. 6.5) (Materials Data, USA) to obtain lattice parameter and size information of crystal. Thermogravimetry (TG) curves of Ag@TiO_2_-PPDOs were obtained from a DTG-60 thermal gravimetric analyzer (Shimadzu, Japan). The concentration of Ag on the surface of Ag@TiO_2_-PPDOs was investigated using Gemini 5000 EDS (Carl Ze ss, Germany), and the metal content (Ag, Ti) of Ag@TiO_2_-PPDOs was detected by Inductively Coupled Plasma (ICP) (Thermo Fisher, USA). UV-DRS and Raman spectrums of Ag@TiO_2_-PPDOs were recorded by ultraviolet spectrophotometer Uv3600 (Shimadzu, Japan) and Raman spectrometer RM3000 (Shimadzu, Japan). Size of Ag@TiO_2_ were observed by transmission electron microscope (TEM) (JEOL-2100 Plus; JEOL, Ltd., Tokyo, Japan).

### Synthesis of Ag@TiO_2_ composites

Considering the preparation of 8% Ag@TiO_2_ composite as an example, the process is presented as follows. First, 25.1 mg of AgNO_3_ was dissolved into 20-mL deionized water while stirring, then 200 mg of TiO_2_ nanoparticles was added into the aforementioned solution and stirred for another 10 min. Finally, the TiO_2_ nanoparticles/AgNO_3_ solution mixture was sonicated for 30 min to obtain a uniform suspension. Then, the suspension was dried at 70 °C to obtain solids after 24 h of culturing. Subsequently, the obtained solids were grinded and heated up to 100 °C with a heating rate of 2 °C/min, and after holding at 100 °C for 20 min, the temperature was raised up to 350 °C and maintained for 5 h to obtain the final 8% Ag@TiO_2_ composite.

### Synthesis of Ag@TiO_2_-PPDOs

First, 100 mg of Ag@TiO_2_-PPDO was added into a flask, then 2 g of p-dioxanone (PDO) was added into flask and 2-mg stannous octoate. The flask was sealed using a long-neck pipe connected to an oil pump to remove oxygen and moisture, and then filled with nitrogen. This process was repeated thrice until the moisture and oxygen in the flask were removed, as much as possible. Then, the flask was sonicated for 30 min at 30 °C until Ag@TiO_2_ could be evenly distributed in the melted PDO monomers. Finally, the gas in the flask was removed by an oil pump and polymerization was conducted under vacuum conditions at 110 °C under magnetic stirring for 24 h. Subsequently, the reaction mixture was dissolved in hexafluoroisopropanol and stirred for 30 min and centrifuged to discard the undissolved Ag@TiO_2_. Absolute ethyl alcohol was dropped into the supernatant liquid to deposit Ag@TiO_2_-PPDO, then the deposits were filtered out and dried under vacuum conditions at 40 °C. Compositions of series of Ag@TiO_2_-PPDOs are listed in Table 1.

**Table 1:** Series of Ag@TiO_2_-PPDOs.

Series	Feed weight ratio of Ag to TiO_2_ nanoparticles	Feed weight ratio of Ag @TiO_2_ to PDO
8% Ag@ TiO_2_ PPDO	8:100	1:20
12% Ag@ TiO_2_ PPDO	12:100	1:20
16% Ag@ TiO_2_ PPDO	16:100	1:20
20% Ag@ TiO_2_ PPDO	20:100	1:20

### Bacteriostatic test

After 18 h of the cell culture, the *Staphylococcus aureus* suspension were diluted to 0.5 McFarland in a sterile nutrient solution. Then, 80 μL of bacteria suspension was uniformly coated over the nutrient in culture dishes. Subsequently, 10 mg, 15 mg, and 20 mg of Ag@TiO_2_-PPDOs were pressed into circles with diameters of 6 mm, 8 mm, and 1 cm, respectively and then placed on the surface of the culture dishes. Furthermore, the *Staphylococcus aureus* and Ag@TiO_2_-PPDO-vaccinated culture dishes were cultured at 37 °C for 24 h. Finally, the bacteriostatic effect of different types of Ag@TiO_2_-PPDOs was evaluated by measuring their bacteriostatic zones.

### Preparation of Ag@TiO_2_-PPDO-coated PLGA electrospun membranes

Ag@TiO_2_-PPDO was added into CH_2_Cl_2_/CH_3_OH (9:1, v:v) to form a stable dispersed system with a concentration of 2% w/v. The coating of Ag@TiO_2_-PPDOs onto PLGA electrospinning membrane was conducted by electrospraying. The PLGA electrospinning membrane was placed on the collected drum, which was connected with negative electrode. Ag@TiO_2_-PPDO suspension was added into a 10-ml syringe, whose needle was connected with a positive electrode. The applied voltages were 9 kV (positive electrode) and − 2.5 kV (negative electrode). The flow rate of the Ag@TiO_2_-PPDO suspension was 0.2 ml/L, while the distance between the needle tip to the PLGA membrane was 10 cm. Finally, the obtained mass ratio of Ag@TiO_2_-PPDO to PLGA membrane was 1:5.

### Animal experiment

In total, 18 rats were randomly divided into three groups, and after the hairs on their backs were removed, the rats were anesthetized using an intraperitoneal injection of a pentobarbital sodium solution (3%, Sigma) at a dosage of 1.0 mL/kg. After local disinfection with a povidone iodine (PVP-I) solution (5%), the sterilized Ag@TiO_2_-PPDOs coating PLGA electrospun membranes (2 mg), PLGA electrospun membranes, and 0.5 ml saline were embedded into the subcutaneous tissue pockets of the rats for the Ag@TiO_2_-PPDOs coating of the PLGA membrane treated group, PLGA membrane treated membrane, and control group. Before the layered suture, 10 μL of *Staphylococcus aureus* suspension with a concentration of 4 × 10^7^ CFU per milliliter was injected into the subcutaneous pockets to mimic infective conditions after the implantation of biomaterials. Then, 7 days post operation, the rats were euthanized, and the skin and subcutaneous tissue at implantable sites, including skin implanted membranes and anadesma, were incised and kept in neutral formalin (10%) for 24 h. The fixed tissues were paraffin-embedded, dehydrated, and sectioned for H&E staining and TNF-α immunohistochemical staining. The average IOD/pixel^2^ was applied to reflect the staining depth and range of TNF-α immumohistochemical staining, which were obtained by the Image Pro Plus 6.0 software based on photographs of five randomly selected fields under 400 × magnification for each section.

### Statistics

Data are expressed as mean ± standard error of the mean. Statistical comparisons were made by one-way analysis of variance. *P* < 0.05 was considered statistically significant.

### Study approval

Animal procedures were approved by the Animal Care and Treatment Committee of the North Sichuan Medical College and performed in accordance with relevant guidelines and regulations of Chinese Association for Laboratory Animal Science for the Care and Use of Laboratory Animals and the ARRIVE guidelines.

### Data availability

The datasets generated and analyzed during the current study are not publicly available at this time due to the data also forms part of an ongoing study but are available from the corresponding author on reasonable request.

## Result and Discussion

### Synthesis of Ag@TiO_2_-PPDO

**Figure 2:**
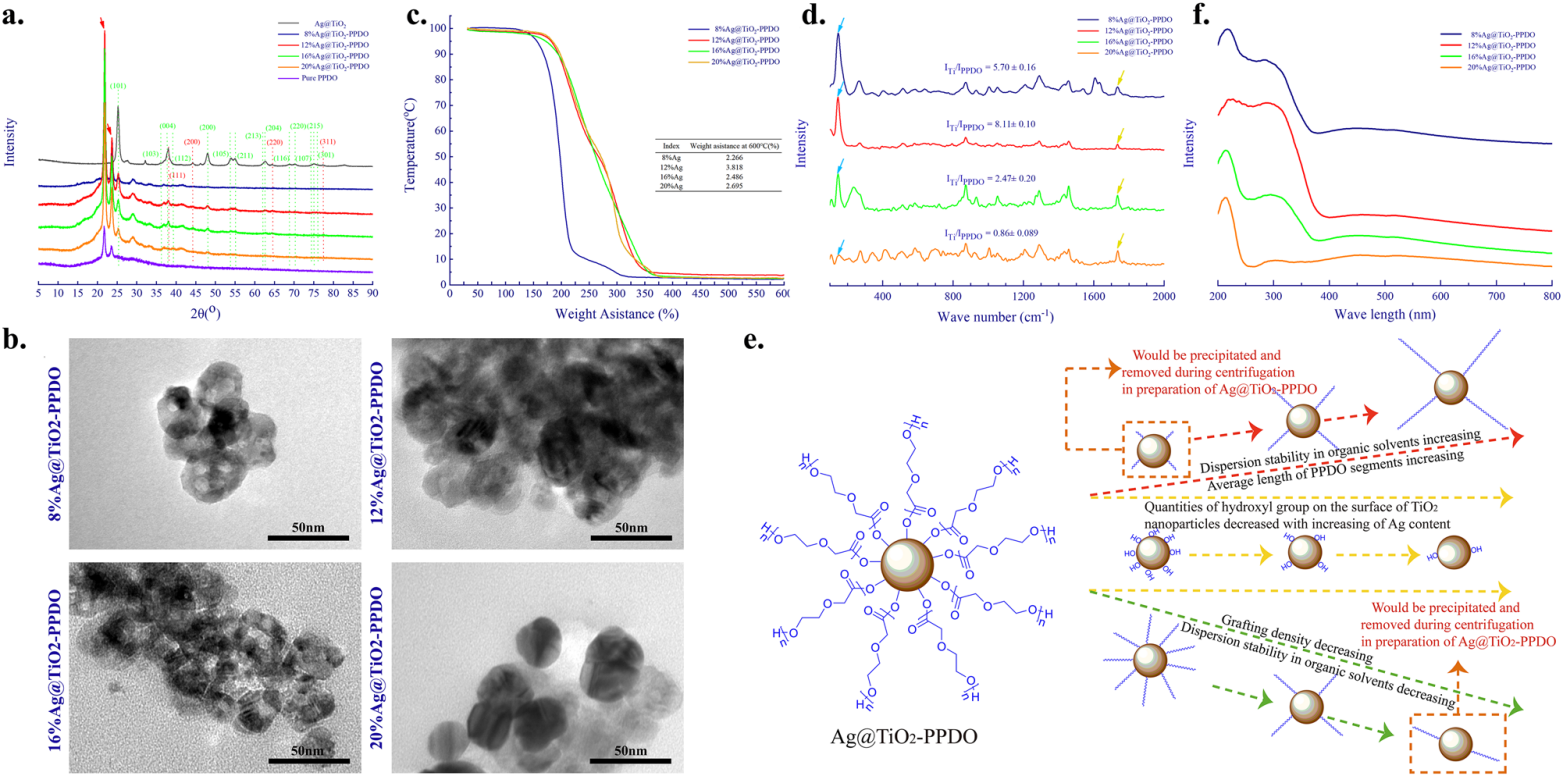
(**a**) XRD spectra of Ag@TiO_2_-PPDOs (red arrows indicates peaks of PPDO crystal, the green dotted line indicates peaks of TiO_2_ crystal, the red dotted line indicates peaks of Ag); (**b**) TEM of Ag@TiO_2_-PPDOs; (**c**) TG curves of Ag@TiO_2_-PPDOs; (**d**) Raman spectrum of Ag@TiO_2_-PPDOs; (**e**) Affection of Ag content in Ag@TiO_2_ on the disperse stability of Ag@TiO_2_-PPDO in organic solvent; (**f**) UV-DRS determination of Ag@TiO_2_-PPDOs.

Because Ag@TiO_2_ nanoparticles were prepared by a hydrothermal process, abundant hydroxyl groups would be left on the surface of Ag@TiO_2_ nanoparticles^36^. Then, as illustrated in Fig. 1, Ag@TiO_2_-PPDOs could be obtained by the polymerization of PDO initiated by hydroxyl on the surface of Ag@TiO_2_ nanoparticles. The XRD spectra of obtained Ag@TiO_2_ nanoparticles, Ag@TiO_2_-PPDOs, and PPDO were presented in Fig. 2a. Diffractive peaks indexed to metal Ag can be found at 2θ = 44.3° (200), 64.2° (220) and 77.5° (311), indicating that the silver was atomically loaded onto TiO_2_^23^. The lattice parameters of Ag@TiO_2_ nanoparticles were calculated as a = 0.504 nm, b = c = 1.05 nm, α = β = γ = 90° and the size of crystallite was about 13 nm. Both the lattice size and crystallite size of Ag@TiO_2_ nanoparticles were larger than those of pure anatase TiO_2_, indicating that Ag was definitely embedded into the crystal defects of TiO_2_^37^. Characteristic 2θ peaks between 20° and 25° that belong to PPDO macromolecules could be observed in the XRD spectra of Ag@TiO_2_-PPDOs, and characteristic 2θ peaks of Ag and TiO_2_ could also be observed^38^. The This indicates that PPDO macromolecules were grafted successfully onto the surface of Ag@TiO_2_ nanoparticles. TEM was applied to determine the size of Ag@TiO_2_-PPDOs nanoparticles. As shown in Fig. 2b the average diameters of Ag@TiO_2_-PPDOs nanoparticles were 30-40 nm, which were larger than the purchased pure TiO_2_ (10-25 nm). Both TiO_2_ phase and grafted polymetric shell layer could be observed obviously. Those results indicates that PPDO has been successfully grafted on to the surface of TiO_2_ particles. Shape and size of Ag@TiO_2_-PPDOs nanoparticles are essential for the clearance of Ag@TiO_2_-PPDOs once the performance of them have fulfilled. Spherical shape and larger size of Ag@TiO_2_-PPDOs making them easy to be caught by mononuclear phagocyte system and then be eliminated from in vivo^39^.

To determine the grafting efficiency of PPDO on Ag@TiO_2_ nanoparticles, TG analyses and Raman spectrum of Ag@TiO_2_-PPDOs were conducted to verify the proportions of inorganic compounds. For Raman spectrum, the ratios of intensity of peak at 147 cm^−1^ (Ag@TiO_2_) to that at 1750 cm^−1^ (PPDO) were applied to indicate the proportions of inorganic phase in Ag@TiO_2_-PPDOs^40,41^. Because Ag@TiO_2_ nanoparticles grafted without or with inadequate PPDO segments were centrifugally discarded during the purification process of Ag@TiO_2_-PPDOs, grafting efficiencies would be increased with the final proportions of inorganic compounds in these composites. As illustrated in Fig. 2c,d, the grafting efficiency of 12% Ag@TiO_2_ was the highest. As Ag was embedded embedded into the crystal defects of TiO_2_ as determined by XRD or possibly present island pattern on the surface of TiO_2_ nanoparticles as shown in Fig. 2b, the quantities of hydroxyl groups on the surface of TiO_2_ nanoparticles would decrease^24^. Subsequently, hydroxyl groups on the surface of TiO_2_ nanoparticles decreased with an increase in the Ag content loaded in TiO_2_ nanoparticles. Hydroxyl groups on the surface of TiO_2_ nanoparticles functioned as initiator to initiate the polymerization of PDO monomers; in other words, hydroxyl groups were the active growth center of PPDO segments^42^. As the active growth center decreases, PPDO grafting density decreases with the quantities of hydroxyl groups. In addition, as the feed molar ratio of PDO to TiO_2_ nanoparticles is fixed, the average segmental length increases with decreasing hydroxyl groups. Then, as illustrated in Fig. 2e, although PPDO grafting density decreases with Ag content in TiO_2_, the average length of PPDO segments increases with Ag content in TiO_2_. However, both the extremely low grafting density and relatively short average length of PPDO segments are not conductive to the dispersion of Ag@TiO_2_-PPDO in organic solvent. Furthermore, Ag@TiO_2_-PPDOs with low grafting density and short PPDO segments were centrifuged out during the purification process of Ag@TiO_2_-PPDOs. Consequently, the maximum grafted efficiency did not always increase or decrease with the Ag loading quantities of TiO_2_ nanoparticles but reached its peak value at a medium Ag loading of 12% Ag@TiO_2_ nanoparticles. The active growth center of 8% Ag@ TiO_2_ nanoparticles was the highest, and the average length of PPDO segments should be the lowest. Then, as illustrate in Fig. 2b, the initial decomposed temperature of organic PPDO segments of 8% Ag@TiO_2_-PPDO was significantly lower than those of the other three types of Ag@TiO_2_-PPDOs.

To determine whether photo-responsive properties of Ag@TiO_2_-PPDOs still present, absorptions of Ag@TiO_2_-PPDOs at UV–visible region were obtained by UV-DRS determinations. As shown in Fig. 2f, all four Ag@TiO_2_-PPDOs have absorptions at UV region. Specifically, absorption of Ag@TiO2-PPDO nanoparticles in the visible light range could also be observed. When TiO_2_ nanoparticles are exposed to UV light (particularly near-ultraviolet) light-photo excited electron (e−) excites from the valence band to the conduction band generating a positively charged hole (h^+^). The generated h^+^ undergoes an oxidation reaction whereas the e^-^ undergoes a reduction processes by reacting with O_2_, H_2_O or OH^-^ to produce ROS, which undergo some reactions to inactivate and oxidize bacteria. The addition of Ag to TiO_2_ and forming composite structures could enhance absorption in the visible light range. The visible light absorption result of the localized surface plasmon response (LSPR) properties of Ag present in Ag@TiO_2_-PPDO nanoparticles^22,43–45^. Besides, incorporating of Ag on the surface of TiO_2_ NPs could create electron trap at their interfaces promoting interfacial charge transfer and preventing recombination of e^–^h^+^ pairs, which also improves antibacterial and self-cleaner activity of TiO_2_^22^.

**Figure 3:**
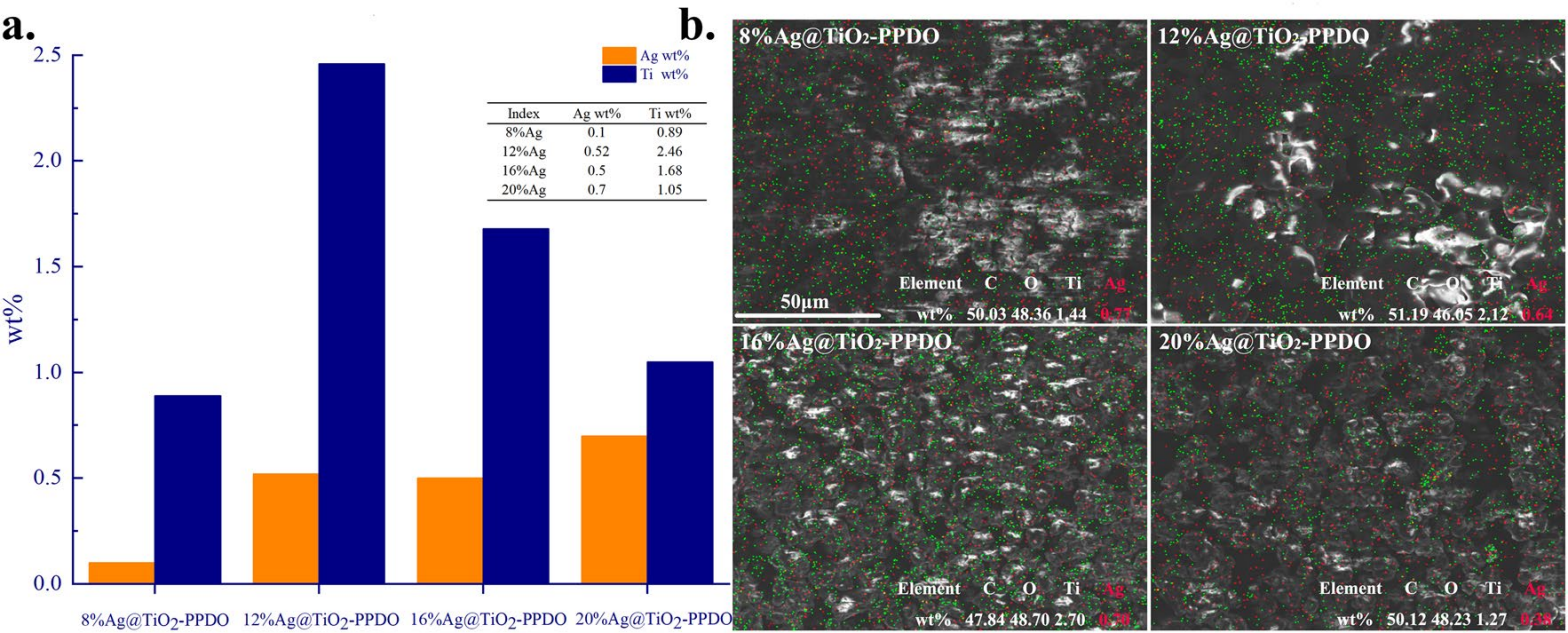
(**a**) Ag content detected by ICP; (**b**) SEM–EDS mapping of Ag@TiO_2_-PPDOs.

Because Ag was the main antibacterial ingredient in the subsequently applied Ag@TiO_2_-PPDOs, the overall Ag contents in Ag@TiO_2_-PPDOs and on the surfaces of Ag@TiO_2_-PPDOs was detected via ICP and SEM–EDS methods. It could be observed from Fig. 3a and that the overall Ag content primarily increased with the feed molar ratio of Ag. The overall Ag content in 12% Ag@TiO_2_-PPDO was slightly larger than that of 16% Ag@TiO_2_-PPDO for that the inorganic Ag@TiO_2_ in 12% Ag@TiO_2_-PPDO was the largest, as illustrated in Fig. 2b. In addition, the overall Ag contents in Ag@TiO_2_-PPDOs and on the surfaces of Ag@TiO_2_-PPDOs decided their initial antibacterial efficiencies. As illustrated in Fig. 3b, the Ag contents on the surface of 12% Ag@TiO_2_-PPDO and 16% Ag@TiO_2_-PPDO were the largest. Although the overall Ag content in 20% Ag@TiO_2_-PPDO was the largest, Ag content on the surfaces of Ag@TiO_2_-PPDOs was not the largest. As shown in Fig. 2c, the average length of PPDO segments on the surface of Ag@TiO_2_ nanoparticles increased with Ag content loaded by TiO_2_ nanoparticles. Then the fairly long of PPDO segments would wrap Ag@TiO_2_ nanoparticles tightly and block Ag presenting on the surface of Ag@TiO_2_-PPDO.

### In vitro antibacterial effects of Ag@TiO_2_-PPDOs

**Figure 4:**
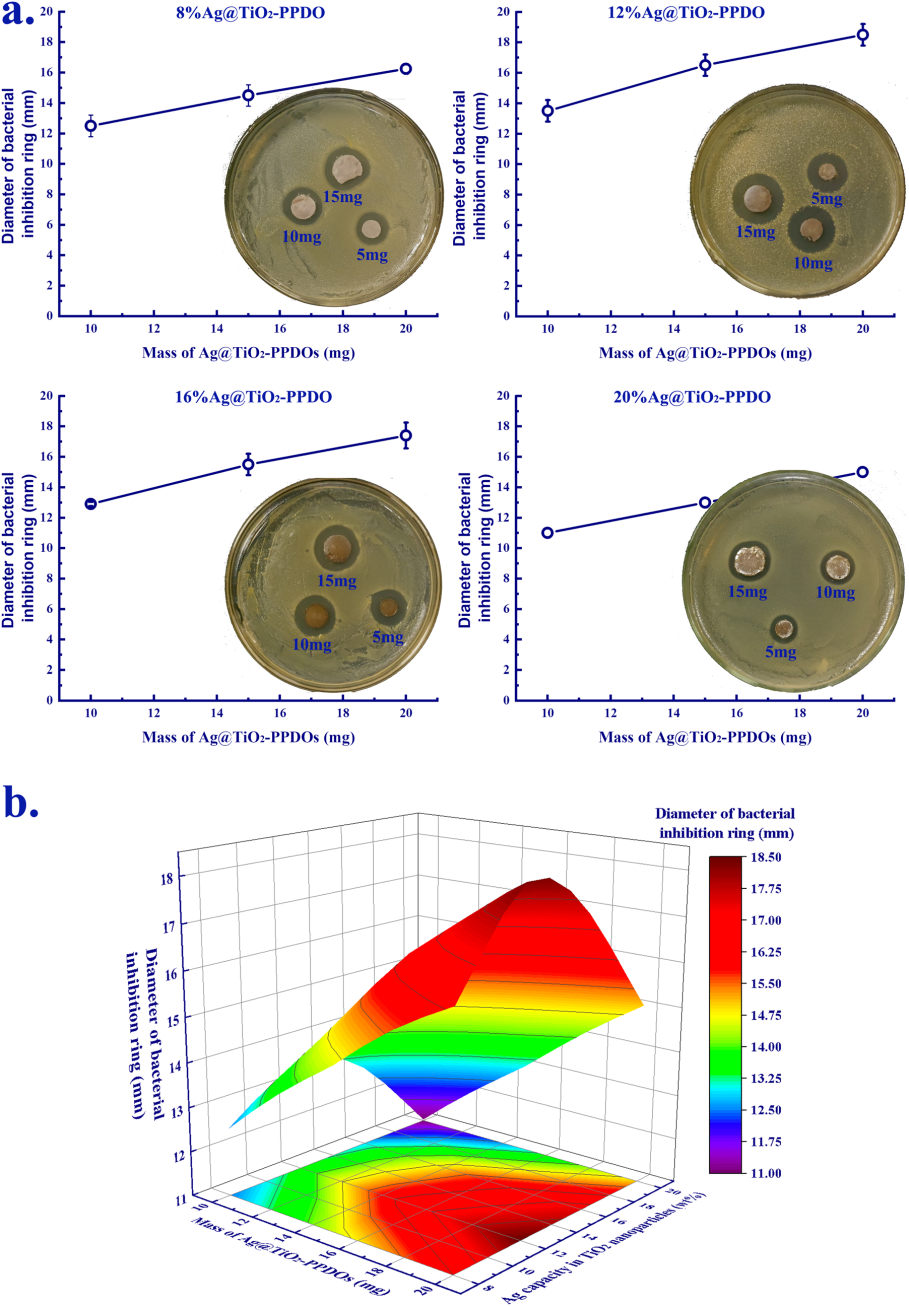
(**a**) Bacteriostatic zone diameter of Ag@TiO_2_-PPDOs with different dosage; (**b**) Correlations of bacteriostatic zone diameter with Ag loading contents and dosage.

The in vitro antibacterial effects of Ag@TiO_2_-PPDOs were assayed by a bacteriostatic zone test. As illustrated in Fig. 4a, all the four types of Ag@TiO_2_-PPDOs could inhibit the growth of bacterial around them, and the bacteriostatic effect was positively correlated with the dosage of Ag@TiO_2_-PPDOs. Subsequently, the correlation of bacteriostatic zone’s diameter among Ag loading quantities and Ag@TiO_2_-PPDOs dosages is presented in Fig. 4b. It can be deduced from Fig. 4b that the 12% Ag@TiO_2_-PPDO maintained the best bacteriostatic effect with its highest dosage. Because the overall Ag contents on the surfaces of 12% Ag@TiO_2_-PPDO and 16% Ag@TiO_2_-PPDO were the highest, the bacteriostatic effects of these two composites should be the best. However, as demonstrated in Fig. 4b, the bacteriostatic effect of 12% Ag@TiO_2_-PPDO was better than that of 16% Ag@TiO_2_-PPDO. This could be attributed to the fact that the average length of PPDO segments of 16% Ag@TiO_2_-PPDO was larger than that of 12% Ag@TiO_2_-PPDO (Fig. 2c). Subsequently, although the Ag content on the surface of 16% Ag@TiO_2_-PPDO was comparable with that of 12% Ag@TiO_2_-PPDO, the sustained release of bacteriostatic Ag^+^ was hindered by the blocked PPDO segments. Then, the 12% Ag@TiO_2_-PPDO with the best bacteriostatic efficiency was selected to conduct the subsequent in vivo anti-infective experiment.

### In vivo bacteriostatic effect of Ag@TiO_2_-PPDOs coating PLGA/PLCA membranes

**Figure 5:**
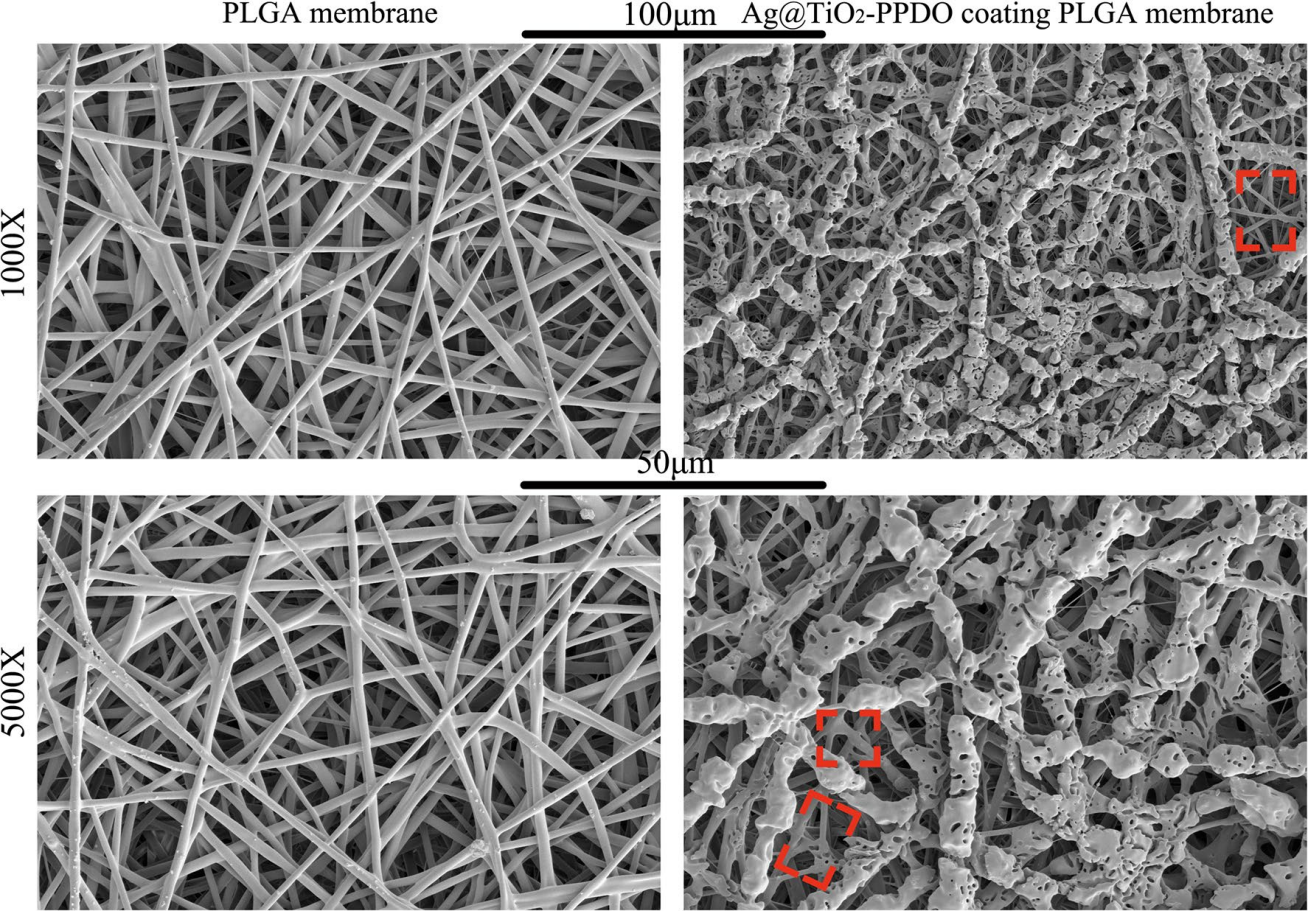
SEM photographs of implanted PLGA electrospun membrane and Ag@TiO_2_-PPDO-coated PLGA membrane. (Red square indicates PLGA membranes under Ag@TiO_2_-PPDO coating).

**Figure 6:**
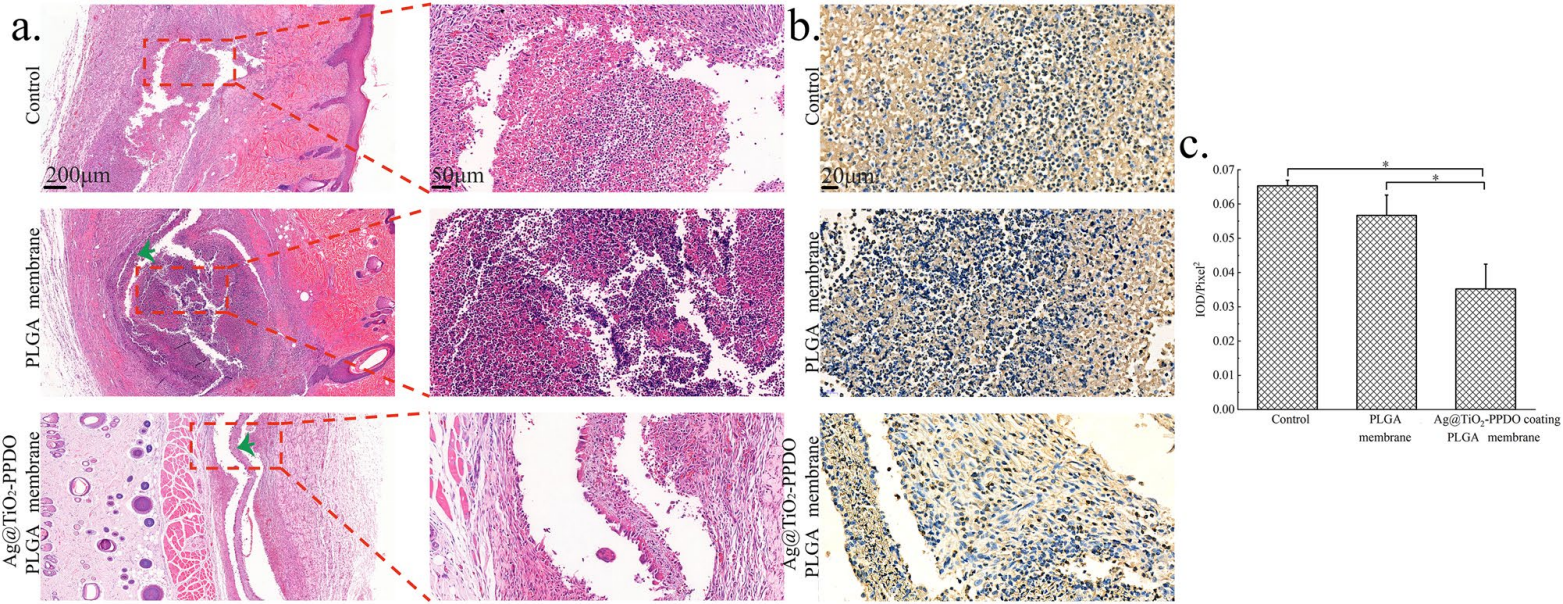
(**a**) H&E staining (green arrows indicate the implanted membranes); (**b**) TNF-α immumohistochemical staining of subcutaneous tissues treated with different materials; (**c**) Statistical IOD of TNF-α immumohistochemical staining.

First, a biodegradable PLGA membrane was fabricated via electrospinning, and then Ag@TiO_2_-PPDOs were coated on the PLGA membrane by electrospraying. As illustrated in Fig. 5, fibrous micro meshes were successfully obtained, while Ag@TiO_2_-PPDOs were coated on the surface of the mesh along the direction of PLGA fibers. Fibrous structures of the PLGA membrane under Ag@TiO_2_-PPDOs coatings have been adequately maintained. The infective rat subcutaneous implanted model was applied to evaluate the in vivo bacteriostatic effect of Ag@TiO_2_-PPDOs coatings. The obtained histological staining results are illustrated in Fig. 6, where severe infections and even abscess cavity and abundant inflammatory cells can be observed in the control and especially PLGA membrane treated groups, owing to the aggravative effect of implants on infections under infective conditions. In contrast, no significant infection and inflammatory cell could be observed in the Ag@TiO_2_-PPDO-coated PLGA membrane treated group, which indicates that the Ag@TiO_2_-PPDO coatings have effectively defected the infection caused by the proliferation of injected bacteria. To further verify the observation in Fig. 6a, TNF-α stanning was applied to evaluate the inflammatory degree. As illustrated in Fig. 6b,c, the TNF-α expression of tissues treated with Ag@TiO_2_-PPDO-coated PLGA membrane was significantly lower than those of the control and PLGA membrane treated groups, which indicates that the application of Ag@TiO_2_-PPDOs coatings has significantly decreased the inflammatory reaction caused by bacterial infections. This result is consistent with the phenomena observed by H&E staining.

Implant-associated infection is a major limitation facing biomaterial implantable therapy that can trigger severe problems ranging from the dysfunction of implanted biomaterials to sepsis in patients^46^. As soon as bacteria from the atmosphere, endogenous migration from other parts of host, and hand of the surgeon attach to the surfaces of implanted materials, the colonization of bacteria leading to the formation of a biofilm occurs immediately, which eventually causes infections^46^. This biofilm could protect bacteria from body fluid and immune substances secreted by immune cells, and this type of infective mechanism causes implant-associated infections that are difficult to handle^47^. Even minute quantities of bacteria or normally non-pathogenic pathogens such as *Staphylococcus epidermidis* would cause implant-associated infections, owing to the present of implanted biomaterials. Hence, inducing anti-infective functions to implanted biomaterials is crucial for the success of implantable biomaterial-assisted therapies^48^. Among various modified methods, surface coating or loading of bacteriostatic Ag-contained composites has been widely investigated, owing to its broad-spectrum antibacterial property, adhesion defective effect on bacteria, and rare susceptibility to drug resistance of Ag ions^49^. However, conventional coating methods might be unsuitable to modify organic biodegradable implantable biomaterials with weak mechanical strength or sensitivities to high temperature and humidity such as electrospun membranes. Electrospun membranes have been increasingly investigated in recent years, owing to their versatile applications in biomedical areas such as their viable functions as anti-adhesive membranes and guided bone regeneration scaffolds^50^. Therefore, in this investigation, biodegradable polymer-grafted Ag@TiO_2_ were designed to aid the stable distribution of Ag@TiO_2_ in organic solvent. Subsequently, Ag@TiO_2_ could be coated on the surface of electrospinning membranes by electrospraying. As demonstrated in Fig. 5, the coating process did not damage the main structure of PLGA/PLCA electrospun membranes. As illustrated in Fig. 6, the pure PLGA membrane implant caused severe implant-associated infections; however, the Ag@TiO_2_-PPDO-coated PLGA membranes significantly eliminated the implant-associated infections, and even original infections. This indicates that the biodegradable polymer-grafted Ag@TiO_2_ coating is a promising antibacterial modifying method for high temperature and humidity sensitive implantable biomaterials.

## Conclusion

Series of PPDO-grafted Ag@TiO_2_ nanoparticles were synthesized successfully by hydroxyl groups that were located on the surface of TiO_2_ nanoparticle-initiated ring opening polymerization of PDO. Among Ag@TiO_2_ nanoparticles with various Ag loading quantities, the 12% Ag@TiO_2_ nanoparticles exhibited the highest grafting efficiency. After grafting, the overall Ag contents of Ag@TiO_2_-PPDOs increased with the Ag loading quantities, and the highest Ag content on the surface of Ag@TiO_2_-PPDOs appeared in both 12% Ag@TiO_2_-PPDO and 16% Ag@TiO_2_-PPDO. In addition, the 12% Ag@TiO_2_-PPDO exhibited the best bacteriostatic effect in vitro, owing to its higher grafted efficiency and relatively short length of PPDO segments. Ultimately, the in vivo bacteriostatic effect of 12% Ag@TiO_2_-PPDO coating was verified. As Ag@TiO_2_-PPDOs were easily processed and could be stably dispersed in the organic solvent; hence, they are promising bacteriostatic coating materials for biodegradable temperatures and humidity-sensitive medical devices.
